# Proceedings from the 2021 SAEM Consensus Conference: Research Priorities for Interventions to Address Social Risks and Needs Identified in Emergency Department Patients

**DOI:** 10.5811/westjem.2022.11.57293

**Published:** 2023-02-25

**Authors:** Liliya Kraynov, Aaron Quarles, Andrew Kerrigan, Katherine Dickerson Mayes, Sally Mahmoud-Werthmann, Callan E. Fockele, Herbert C. Duber, Kelly M. Doran, Michelle P. Lin, Richelle J. Cooper, Nancy Ewen Wang

**Affiliations:** * Valleywise Health, Department of Emergency Medicine, Phoenix, Arizona; † Northwestern University Feinberg School of Medicine, Department of Emergency Medicine, Chicago, Illinois; ‡ University of Southern California, Keck School of Medicine, Department of Emergency Medicine, Los Angeles, California; § Harvard Medical School, Department of Emergency Medicine, Boston, Massachusetts; ¶ NYU School of Medicine, Departments of Emergency Medicine and Population Health, New York, New York; || Stanford University, Department of Emergency Medicine, Stanford, California; # University of Washington, Department of Emergency Medicine, Seattle, Washington; ** Icahn School of Medicine at Mount Sinai, Department of Emergency Medicine, New York, New York; †† David Geffen School of Medicine at UCLA, UCLA Department of Emergency Medicine, Los Angeles

## Abstract

**Introduction:**

Emergency departments (ED) function as a health and social safety net, regularly taking care of patients with high social risk and need. Few studies have examined ED-based interventions for social risk and need.

**Methods:**

Focusing on ED-based interventions, we identified initial research gaps and priorities in the ED using a literature review, topic expert feedback, and consensus-building. Research gaps and priorities were further refined based on moderated, scripted discussions and survey feedback during the 2021 SAEM Consensus Conference. Using these methods, we derived six priorities based on three identified gaps in ED-based social risks and needs interventions: 1) assessment of ED-based interventions; 2) intervention implementation in the ED environment; and 3) intercommunication between patients, EDs, and medical and social systems.

**Results:**

Using these methods, we derived six priorities based on three identified gaps in ED-based social risks and needs interventions: 1) assessment of ED-based interventions, 2) intervention implementation in the ED environment, and 3) intercommunication between patients, EDs, and medical and social systems. Assessing intervention effectiveness through patient-centered outcome and risk reduction measures should be high priorities in the future. Also noted was the need to study methods of integrating interventions into the ED environment and to increase collaboration between EDs and their larger health systems, community partners, social services, and local government.

**Conclusion:**

The identified research gaps and priorities offer guidance for future work to establish effective interventions and build relationships with community health and social systems to address social risks and needs, thereby improving the health of our patients.

## BACKGROUND

Although the concept of social medicine has existed for nearly two centuries, the contemporary medical community has only more recently acknowledged the interconnectedness of socioeconomic status and health. Often credited as the founder of social medicine, physician Rudolf Virchow in 1848 helped establish the newspaper *Medical Reform* and brought attention to the social origins of illness.^1^,^2^ More recently, multiple medical organizations, including the American College of Physicians,^3^ the American Academy of Pediatrics,^4^ and the American Academy of Family Physicians,^5^ have advocated addressing social risks and needs in clinical settings to improve health outcomes.

Patients with unmet social risks and needs, such as food insecurity or unstable housing, have a higher prevalence of depression, diabetes, and hypertension, among other health issues.^6^ Children with unmet social risks and needs have a higher prevalence of disease, such as asthma,^7^,^8^ and have worse control of conditions such as type 1 diabetes.^9^ These children are also more likely to experience obesity, diabetes, and cardiovascular disorders in adulthood.^10^ Those with multiple social risks and needs experience a cumulative effect on their health.^11^–^13^

Emergency departments (ED) function as a health and social safety net,^14^,^15^ regularly taking care of patients with high social risks and needs.^16^ Nearly one in four ED patients is food insecure, and one in five reports choosing between food and medication.^17^ Patients seen in the ED experience a high prevalence of financial insecurity,^18^ unreliable transportation,^19^ unemployment,^20^,^21^ and housing instability.^21^,^22^ Visits to the ED present unique opportunities to intercede and address the social risks and needs of patients. Most of the emergency medicine (EM) literature on social determinants of health focuses on identifying and screening for social risks and needs.^16^ Few studies have examined ED interventions to address social risks and needs. In this article, we describe the research gaps and priorities for interventions addressing social risks and needs identified as part of the 2021 Society for Academic Emergency Medicine (SAEM) Consensus Conference – From Bedside to Policy: Advancing Social Emergency Medicine and Population Health through Research, Collaboration, and Education.

## METHODS

The leadership team of the 2021 SAEM Consensus Conference session on social risks and needs screening identified three topics for review: 1) instruments used for social risks and needs screening in the ED; 2) implementation of social risks and needs screening in the ED; and 3) interventions for patients with social risks and needs in the ED.^23^ In this paper we address the third topic, presenting gaps in current knowledge and research priorities focused on interventions for patients with identified social risks and needs. For consistency across these three topics, we have adopted the definitions for social determinants of health as per Alderwick et al: social risk, defined as social conditions associated with poor health; and social need, defined as these social conditions with which patients would like assistance in addressing.^24^

**Population Health Research Capsule**

What do we already know about this issue?

*Emergency departments (ED) serve as a safety net by regularly taking care of patients with high social risks and unmet social needs*.

What was the research question?

*What are the research gaps and priorities in interventions for ED patients with social risks/needs?*

What was the major finding of the study?

*We identified three gaps and six research priorities in ED-based social risks and needs interventions*.

How does this improve population health?

*The derived gaps and priorities offer guidance for future research to establish effective ED-based interventions and build links between health and social systems*.

### Literature Review

We conducted a literature review building upon a previously published systematic review on ED patients’ social risks and needs.^16^ With the assistance of a health sciences librarian, we used a PubMed search strategy that identified 2,085 articles across the three objectives (Appendix A). A review of titles and abstracts resulted in 151 potentially relevant articles across the continuum from screening through interventions. We complemented the PubMed search with a review of the Social Interventions Research and Evaluation Network (SIREN) Evidence and Resource Library, which compiles research on medical and social care integration.^25^ Based on titles and abstracts, authors HD and CF identified an additional 22 potentially relevant articles. Of the 173 total manuscripts identified, 18 applied to our topic—interventions for identified social risks and needs—after review of the full article.

**Figure 1:**
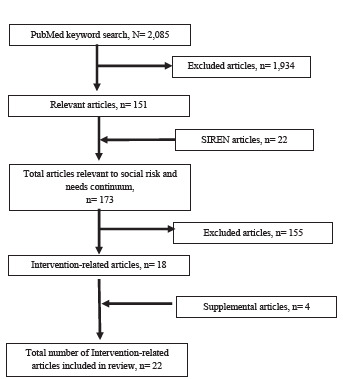
Flow diagram of literature review search results. *SIREN*, Social Interventions Research and Evaluation Network.

We excluded articles if they had not been conducted in the ED or an urgent care within a hospital. Articles with interventions conducted across a hospital or health system, even if they did not focus primarily on ED patients, were included if the intervention was also incorporated into the ED. We then supplemented our article searches by checking the references within these 18 publications for additional pertinent articles to our topic; we identified four additional articles. In total, 22 articles were included in our review (Figure 1).^26^–^47^

### Initial Derivation of Research Gaps and Priorities

For each included study, we extracted data pertaining to study objective, design, outcomes, results, limitations, and noted study quality and risk of bias issues. This data was summarized in an analysis matrix (Microsoft Excel for Mac, version 16.52 (Microsoft Corporation, Redmond, WA). Our group thematically analyzed data from the analysis matrix; we then identified research gaps and drafted preliminary research priorities. We shared the draft research priorities with external expert reviewers from the Department of Health and Human Services Office of the Assistant Secretary for Planning and Evaluation,^48^ Health Leads,^49^ and SIREN,^50^ incorporating their feedback into a document outlining preliminary research gaps and priorities (Appendix B).

### Consensus-building and Derivation of Final Research Gaps and Priorities

**Figure 2:**
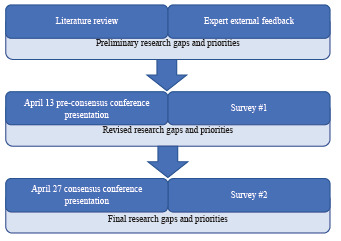
Consensus process to identify social risks and needs interventions.

The SAEM Consensus Conference was convened in two sessions virtually over Zoom (Zoom Video Communications, Inc, San Jose, CA) on April 13 and 27, 2021 (Figure 2). Preliminary research gaps and priorities (Appendix B) were presented to participants of the Consensus Conference during the moderated first session on April 13. Conference participants included academic EM faculty and residents, community emergency physicians, and medical students. Then, scripted moderated discussions followed based on the previously identified gaps. Participants were allowed time to give verbal feedback. After the presentation session, registered conference participants provided feedback using an electronic survey (Table 1). A free-text option was included in the survey.

The survey questions were developed and distributed by the Consensus Conference leadership for each objective subgroup. Survey feedback was incorporated into a revised list of research priorities, and the revised list was presented in small groups during session two of the SAEM 21 Consensus Conference on April 27. Participants were then sent a second survey asking them to rank what they believed were the top three research priorities for social risks and needs interventions in the ED. Priorities were scored and then ranked, using the following formula:


$$\text{Total score}=3\text{x }(\#\;1^{\text{st}}\;\text{choice votes})+2\text{x }(\#\;2^{\text{nd}}\;\text{choice votes})+1\text{x }(\#3^{\text{rd}}\;\text{choice votes}).$$

Priorities were ranked as high, medium, or low based on the top one-third, middle one-third, and lowest one-third of votes, respectively (Table 2).

## FINDINGS and DISCUSSION

Overall, our workgroup identified 22 studies evaluating social risks and needs interventions among ED patients.^26^–^47^ Initial group discussions identified an abundance of gaps and unanswered questions. We elected to group these gaps into generalized, broad categories rather than focus on granular issues that would not address the breadth of our objective.

Of the 22 studies, one was a systematic review,^42^ five were randomized control trials (RCT) or secondary analyses of an RCT,^29^,^33^–^35^,^43^ while the rest were observational studies. Study size ranged from 19 to 34,225 with most studies including several hundred participants. We identified two studies performed at a non-academic community hospital; the remaining 20 studies were conducted at academic centers.^41^,^45^ Eight studies explicitly mentioned including non-English speaking patients; of these studies, Spanish was the predominant non-English language.^30^,^33^–^35^,^39^,^43^,^44^,^46^ Nine studies did not explicitly state whether they included non-English speakers.^26^–^28^,^32^,^36^,^40^,^41^,^45^,^47^ Only one study included a rural site.^32^

### Gap 1: Assessing Intervention Effectiveness

Our literature review revealed a variety of outcome measures used to evaluate intervention performance. Twelve studies relied on the number of referrals placed to community resources,^26^–^29^,^36^–^42^,^47^ six reported community resource utilization,^26^,^29^,^35^,^39^,^44^,^47^ six reported healthcare utilization,^27^,^39^,^43^–^46^ and only one analyzed cost savings.^44^ Four studies described patient satisfaction with the intervention,^26^,^28^,^39^,^41^ and six presented self-reported health improvements as outcomes.^26^,^32^,^34^,^37^,^38^,^42^ Our group discussions noted a lack of patient-centered outcomes in past studies. Expert comments, discussions during the Consensus Conference, and survey results agreed that identifying appropriate patient-centered outcomes, such as hunger-free days, improvement in housing, and symptom reduction should be a high research priority in the future.

We noted a literature gap in evaluating intervention cost and cost savings for patients and healthcare systems. One of our expert reviewers agreed that this should be an area of future exploration. Another expert reviewer noted that cost savings would be challenging to measure (eg, secondary to cost-shifting), and research surrounding cost may prematurely divert attention from examining the efficacy of the interventions. As cost is generally not a patient-centered outcome and is borne by the healthcare system or insurers, and because our goal is to improve the health and quality of life for patients, our workgroup chose to prioritize questions related to intervention effectiveness, rather than cost.

The initial research priorities included a question regarding the hypothesized time horizon for evaluating the impact of interventions, given concern that time frames for seeing impact from interventions addressing social needs might be longer than examined in most traditional medical studies. This question was presented during the first session on April 13, ranked low in the first survey, and did not receive any votes in the final survey. We ultimately did not include this question separately in the final research priorities, but a consideration of timeframe is inherent in the questions evaluating intervention effectiveness.

We identified only four comparative effectiveness studies of social need interventions.^33^–^35^,^43^ Three separate questions were initially presented during the Consensus Conference addressing the comparative effectiveness of interventions. All three ranked highly in the first survey. Based on discussions during the conference, we combined these into question 2 below, which also rated as high priority in the final survey.

The following research priorities were developed to address the assessment of interventions:

1. Which patient-centered outcomes (eg, resolution of social need, patient self-identified need or improvement, health metrics) should be used to assess the impact of interventions?
2. Which interventions are more effective in reducing social risk and helping address patients’ social needs? Which interventions are not effective and should be abandoned?

### Gap 2: Integration of Interventions into the ED Environment

Our literature review revealed that while some studies have examined interventions in practice and comment on implementation, no study has sought to evaluate implementation rigorously. While implementation strategies will vary based on location, studies examining the operationalization of interventions can guide the uptake and maintenance of interventions in other EDs.

Many questions regarding logistical barriers and catalysts to implementation remain. For instance, who should deliver the intervention (eg, physician, nurse, social worker, case manager, patient navigator)? Our literature review found that social workers, case managers, and resource navigators tended to be responsible for implementing ED-based social needs interventions.^26^,^27^,^30^,^33^–^35^,^37^,^38^,^40^–^46^ No study directly compared the uptake of an intervention based on whether members of the clinical team (eg, physicians, nurses) or ancillary staff (eg, social workers, case mangers) delivered the intervention. Expert reviewers emphasized the need to assess which staff should be involved and how interventions should be structured. Participants also emphasized staffing limitations as a barrier to uptake and the need for support staff to be included in future research designs and methods.

Studies examining the timing of the intervention during the ED visit (eg, waiting room, in the exam room, post-ED visit), the burden of intervention documentation, how the intervention affects length of stay, and whether the intervention increases task burden will be essential for the uptake of and adherence to the intervention. After incorporating all feedback, the final research priorities are as follows, with the first ranking medium priority and the second ranking high priority:

1. How can EDs reduce barriers (eg, clinician/staff burnout, ED length of stay, electronic health record (EHR)/documentation burden) and increase acceptance of interventions?
2. How can EDs integrate interventions into ED operations to increase feasibility and sustainability? Are existing staffing models sufficient to support the pragmatic implementation of interventions?

### Gap 3: Engagement with Medical and Social Systems

The final research gap, engagement with medical and social systems, arose during conference discussions on the use of technology in interventions. The initial gap and associated research questions proposed by our workgroup focused on different technology used in interventions (Appendix B). Our literature review found that most interventions relied on phone calls, made either by patients or non-clinical staff, to link patients with resources.^26^,^27^,^35^,^37^,^38^,^41^,^43^–^46^ Four studies reported interventions integrated into the EHR in some manner.^27^,^40^,^44^,^45^ Two studies examined the benefit of using texting for linkage to community resources.^28^,^46^ However, expert reviewers were more interested in whether interventions linked patients with resources, as well as EDs with larger health and social systems, rather than the technology used for linkage. For example, they felt it was more important to know that an intervention establishes communication between the ED and the organization providing services to patients rather than whether they used phone calls, faxing, a phone app, EHR referrals, or another form of technology.

Like the expert reviewers, participants in the conference discussion highlighted the need for good communication between patients and medical or social resources, and between the ED and other community resources (eg, food banks, shelters), the larger health system (eg, primary clinics, pediatric clinics), emergency medical services (EMS), and local government. Again, the emphasis was more on facilitating communication between stakeholders, rather than the technology itself. One participant commented that while EDs present an opportunity to address social needs, EDs do not exist in a silo; interventions will not succeed without buy-in from and communication with the larger health and social systems. These discussions led to a revision of our initial technology-focused questions into communication-focused questions:

1. How can interventions be tailored to increase patient linkage with resources and facilitate monitoring of outcomes? What forms of technology may be useful?
2. Which interventions increase communication, coordination, and collaboration between EDs, their larger hospital or health systems, EMS, community partners, social services, local government, and other systems? How can EDs provide warm handoffs to these systems?

## CONCLUSION

While the medical community has more recently recognized and advocated for addressing social risk and needs in clinical settings, research regarding interventions for ED patients is scarce. Work during the 2021 SAEM Consensus Conference identified and prioritized gaps regarding intervention outcome measures, implementing interventions in the busy ED environment, and communication between and within health and social systems. The research gaps and priorities identified during the Consensus Conference offer guidance for further work to establish effective interventions and build relationships with community health and social systems to reduce the social risk and address the social needs of our patients.

## Supplementary Information



## Figures and Tables

**Table 1:** Survey questions regarding proposed initial research gaps and priorities.

Are there any research priorities that you feel are missing from this list? Yes/No. (Mandatory)
a. If yes, please list them and note why they should be added. (Optional)
Are there any research priorities that you feel should be removed? Yes/No. (Mandatory)
Which research priorities should be discussed further in the April 27 breakout sessions? Why? (Mandatory)
Please rank the top 3 research priorities based upon their priority for future research. Please consider the SMART criteria (Specific, Measurable, Attainable, Relevant, Time-based) when completing this exercise.

**Table 2:** Ranked research priorities related to interventions addressing social risks and needs among ED patients. Total score is weighted (3 points for priority 1 vote, 2 points for priority 2 vote, and 1 point for priority 3 vote).

Question	Priority 1	Priority 2	Priority 3	Total Points	Priority
Which patient-centered outcomes (e.g., resolution of social need, patient self-identified need or improvement, health metrics, and ED utilization) should be used to assess the impact of interventions?	10	2	7	41	High
Which interventions are most effective in reducing social risks and helping address patients’ social needs? Which interventions are not effective and should be abandoned?	9	4	6	41	High
How can EDs integrate interventions into ED operations to increase feasibility and sustainability? Are existing staffing models sufficient to support the pragmatic implementation of interventions?	4	9	5	35	High
How can EDs reduce barriers (e.g., clinician/staff burnout, ED length of stay, and EHR/documentation burden) and increase acceptance of interventions?	7	3	2	29	Medium
Which interventions increase communication, coordination, and collaboration between EDs, their larger hospital or health systems, EMS, community partners, social services, and other systems? How can EDs provide warm handoffs to these systems?	1	7	5	22	Medium
How can interventions be tailored to increase patient linkage with resources and facilitate monitoring of outcomes? What forms of technology may be useful?	1	5	4	17	Medium
How can interventions effectively leverage the EHR (e.g., the inclusion of ICD-10 codes for social risks/needs in patient problem lists and EHR-facilitated interventions such as auto-referral lists)?	0	4	2	10	Low
Which interventions are favored by patients, clinicians, and hospitals/healthcare systems?	2	0	3	9	Low
What is an adequate length of time to examine social need/risk intervention outcomes? How should we define “short-term” vs “long-term” outcomes?	0	0	0	0	Low

*ED*, emergency department; *EHR*, electronic health record; *EMS*, emergency medical services; *ICD-10*, International Classification of Diseases, 10th Revision.
