# Supplementary material for: Proceedings from the 2021 SAEM Consensus Conference: Research Priorities for Interventions to Address Social Risks and Needs Identified in Emergency Department Patients
Source: West J Emerg Med. 2023 Feb 25;24(2):295–301. doi: 10.5811/westjem.2022.11.57293 (PMC10047718; doi:10.5811/westjem.2022.11.57293)
Supplement: Supplementary file 1 [file wjem-24-295-s001.docx]

**Appendix**

**Appendix A**. Literature Review Search Terms across the continuum of ED social risks and needs screening

|  |  |
| --- | --- |
| SDOH general | ("socioeconomic factors" [Majr:noexp]  “social conditions”[Majr]  "social change" [Majr]  "social class" [Majr]  "social environment" [Majr]  "family characteristics" [Majr]  "Social Determinants of Health" [Mesh]  "Social Environment" [Mesh]  "Health Status Disparities" [Mesh]  "social needs" [tiab]  social determinant*[tiab]  "material needs" [tiab]  social deprivation*[tiab]  social risk*[tiab]  structural vulnerability[tiab]) |
| built environment | ("Built Environment"[Mesh]  built environment[tiab]) |
| childcare | ("Child Care"[Mesh]  Childcare[tiab]  Child care[tiab]  Daycare[tiab]) |
| criminal justice | (Criminal Justice[tiab]  justice involved[tiab]  justice system involved[tiab]  criminal record*[tiab]  criminal history[tiab]  prison[tiab]) |
| discrimination | ("Social Discrimination"[MeSH Terms]  "Prejudice"[MeSH Terms]  "Social Marginalization"[MeSH Terms]  "Social Stigma"[MeSH Terms]  "marginalization"[Title/Abstract]  "Homophobia"[Title/Abstract]  "Racism"[Title/Abstract]  "Sexism"[Title/Abstract]  "race bias"[Title/Abstract]  "racial bias"[Title/Abstract]  "implicit bias"[Title/Abstract]  "explicit bias"[Title/Abstract]  "covert bias"[Title/Abstract]  "ethnic bias"[Title/Abstract]  "gender bias"[Title/Abstract]  "sex bias"[Title/Abstract]  "age bias"[Title/Abstract]  "religious bias"[Title/Abstract]  "weight bias"[Title/Abstract]  "race discrimination"[Title/Abstract]  "racial discrimination"[Title/Abstract]  "ethnic discrimination"[Title/Abstract]  "gender discrimination"[Title/Abstract]  "sex discrimination"[Title/Abstract]  "age discrimination"[Title/Abstract]  "religious discrimination"[Title/Abstract]  "sexuality discrimination"[Title/Abstract]  "weight discrimination"[Title/Abstract]) |
| economic security | (income [majr:noexp]  poverty [majr]  medical indigency [majr]  "Working Poor"[Mesh]  low income[tiab]  financial toxicity[tiab]  financial hardship[tiab]) |
| education/literacy | ("Literacy"[Mesh]  "Educational Status"[Mesh]  "Academic Failure"[Mesh]  Literacy[tiab]  Illiteracy[tiab]  Educational achievement*[tiab]  Educational status[tiab]  Academic failure*[tiab]  Academic success*[tiab]  reading level[tiab]) |
| employment | (employment [Majr]  unemployment [Majr]  return to work [Majr]  Precarious Employment[tiab]  Marginal Employment[tiab]  Employment Insecurit*[tiab]  underemploy*[tiab]  unemploy*[tiab]) |
| food/hunger | (hunger [majr:noexp]  malnutrition [tiab]  malnourished [tiab]  food assistance [majr]  food insecur*[tiab]  "hunger"[tiab]  “Malnutrition"[Mesh]  "Food Assistance"[Mesh]  "Food Supply"[Mesh]  “Supplemental Nutrition Assistance Program”[tiab]  “Women, Infants, and Children Program”[tiab]  Food Stamp*[tiab]  Nutritional Deficienc*[tiab]  Undernutrition[tiab]  Malnourishment[tiab]  Food Security[tiab]  Food scarcity[tiab]  Food aid[tiab]  Food pantr*[tiab]  food bank*[tiab]  Emergency food[tiab]  Food shelf[tiab]) |
| health care access | (("Medical Assistance"[Mesh] AND (social determinant*[tiab] OR "Social Determinants of Health"[Mesh]))  "Medically Uninsured" [Mesh]  "Health Services Accessibility" [Mesh]  "Health services availability" [tiab]  "health care availability" [tiab]  "healthcare availability" [tiab]  "Health services accessibility" [tiab]  "health care accessibility" [tiab]  "healthcare accessibility" [tiab]  "Health services access" [tiab]  "health care access" [tiab]  "healthcare access" [tiab]  "access to Health services" [tiab]  "access to health care" [tiab]  "access to healthcare" [tiab]  "Healthcare Disparities" [Mesh]  Health Care Inequalit*[tiab]  Healthcare inequalit*[tiab]  Health Care Disparit*[tiab]  Healthcare Disparit*[tiab]  Medicaid[tiab]  Uninsured[tiab]) |
| housing quality | (housing [majr:noexp]  public housing [majr]  "housing" [tiab]  "Residence Characteristics"[Mesh]) |
| housing stability | (homeless persons [Majr]  homeless youth [Majr]  "homeless"[tiab]  Homelessness[tiab]  housing stability[tiab]  housing instability[tiab]  Unhoused[tiab]  stable housing[tiab]  unstable housing[tiab]  housing status[tiab]  Evict*[tiab]  Shelter[tiab]  Unsheltered[tiab]) |
| immigration | ("Emigration and Immigration"[Mesh]  "Emigrants and Immigrants"[Mesh]  "Transients and Migrants"[Mesh]  immigration status[tiab]  undocumented[tiab]  Immigrant*[tiab]  Citizenship[tiab]  Migrant*[tiab]) |
| legal services | ("Legal Services"[Mesh] OR legal services[tiab] OR legal representation[tiab] OR Legal advoca*[tiab]) |
| public benefits | ("Public Assistance"[Mesh:noexp]  "Old Age Assistance"[Mesh]  "Social Security"[Mesh]  SSI[tiab]  public aid[tiab]  public assistance[tiab]  public benefits[tiab]  government aid[tiab]  government assistance[tiab]  government benefits[tiab]  Safety net program*[tiab]  social safety net[tiab]) |
| social support/social isolation | ("Social Support"[Mesh]  "Community Networks"[Mesh]  "Social Networking"[Mesh]  "Social Isolation"[Mesh]  "Homebound Persons"[Mesh]  social support[tiab]  community network*[tiab]  social network*[tiab]  social isolation[tiab]  socially isolated[tiab]  Social connection*[tiab]  Homebound[tiab]  Home-bound[tiab]  Housebound[tiab]  House-bound[tiab]  Shut-in*[tiab]  social vulnerability[tiab]) |
| transportation | (((lack*[tiab] OR availa*[tiab] OR access*[tiab] OR obtain*[tiab]) AND transportation[tiab]) OR Car ownership[tiab]) |
| utilities | ((Utilities[tiab] OR Water[tiab] OR Electricity[tiab] OR Heat*[tiab] OR Fuel[tiab] OR gas[tiab])  AND  (insecurity[tiab] OR “cut off” [tiab] OR “turned off” [tiab] OR Disconnect*[tiab] OR Nonpayment[tiab] OR Problems[tiab] OR assistance[tiab])) |
| violence/safety | ((“Violence”[Mesh]  Domestic violence[tiab]  family violence[tiab]  Intimate partner violence[tiab]  Intimate partner abuse[tiab]  Child abuse[tiab]  Child mistreatment[tiab]  Child maltreatment[tiab]  Child neglect[tiab]  Elder abuse[tiab]  Elder neglect[tiab]  Aged abuse[tiab]  spousal abuse[tiab]  partner abuse[tiab]  wife abuse[tiab]  husband abuse[tiab]  physical abuse[tiab]  gun violence[tiab]  physical violence[tiab]  physical abuse[tiab]  Assault*[tiab]  Personal safety[tiab]  Unsafe[tiab]  victim[tiab])  NOT "Workplace Violence" [Mesh] NOT "Workplace Violence") |

**Appendix B.** Initial research gaps and priorities presented during April 13, 2021 pre-consensus conference presentation with post-presentation rankings regarding interventions to address social risks and needs in emergency department patients

| Identified research gaps | Post-presentation ranking/priority |
| --- | --- |
| What patient-level outcomes (e.g., resolution of social need, health metrics, quality of life, improved health, ED utilization, lessening, or the resolution of disparities) should be used to assess the impact of interventions? | High priority  22 votes |
| Over what time period should we examine outcomes for social needs interventions? How should we define "short-term" vs. "long-term" outcomes? | Low priority  2 votes |
| How should we measure the effects of social needs interventions on ED operations (e.g., clinician/staff burnout, ED length of stay, and EHR/documentation burden) to better understand how to best implement such interventions? | Mid priority  8 votes |
| How should interventions (e.g., the inclusion of ICD-10 codes for social risks/needs in patient problem lists and EHR-facilitated interventions such as auto-referral lists) use the EHR? | Mid priority  8 votes |
| What other forms of technology might be useful for social needs interventions? Most currently studied interventions use the telephone for referrals and contacting patients; can other platforms (e.g., text, phone applications) increase patient linkage with resources and facilitate monitoring outcomes? | Low priority  6 votes |
| How can interventions be tailored to best serve patients who may be hard to contact after the ED visit (e.g., because they do not have a phone or do not follow-up in primary care)? | Low priority  4 votes |
| What technology can EDs, health systems, and community partners implement to coordinate social needs care with other providers or systems? | Low priority  4 votes |
| Which types of interventions are most effective in reducing social risks and helping address patients' social needs? What interventions are not effective and should be abandoned? | High priority  16 votes |
| What types of ED-based interventions are most desired from the patient perspective? From the provider's perspective? From the hospital/systems perspective? | Low priority  6 votes |
| What are the comparative effectiveness and value of interventions that seek to address multiple social risk/need domains simultaneously versus single risk/need domains judged to be most critical for the specific ED setting (e.g., those that are most prevalent or have the largest effects on certain metrics)? | Low priority  2 votes |
| What is the comparative effectiveness of interventions targeted based on social risk screening versus provision of resources in the ED/hospital (e.g., a referral to a food bank vs. an on-site hospital food bank)? | Low priority  4 votes |
| Who should be involved in delivering ED-based social need interventions (e.g., volunteers, research navigators, case managers, community health workers, clinicians)? What interventions can be provided effectively by existing ED staff (e.g., physician or nurse staff, ED social workers) versus additional specialized staff (e.g., resource navigators)? | Low priority  2 votes |
| How can EDs collaborate with their larger hospital or healthcare system, community organizations, social services, or other systems that are more equipped for longer-term follow-up and engagement? How can EDs provide warm handoffs to these systems? | Mid priority  12 votes |

**Appendix C:** Revised research gaps and priorities for Interventions to address social risks and needs in emergency department patients after consensus conference discussion, and ranking on final survey

| Gap 1: Assessing intervention effectiveness | |
| --- | --- |
| Which patient-centered outcomes (e.g., resolution of social need, patient self-identified need or improvement, health metrics, and ED utilization) should be used to assess the impact of interventions? | High priority  19 votes |
| What is an adequate length of time to examine social need/risk intervention outcomes? How should we define "short-term" vs. "long-term" outcomes? | Low priority  0 votes |
| Which interventions are most effective in reducing social risks and helping address patients' social needs? Which interventions are not effective and should be abandoned? | High priority  19 votes |
| Which interventions are favored by patients, providers, and hospitals/healthcare systems? | Low priority  5 votes |
| Gap 2: Integration of interventions into the ED environment | |
| How can EDs reduce barriers (e.g., clinician/staff burnout, ED length of stay, and EHR/documentation burden) and increase acceptance of interventions? | Mid priority  12 votes |
| How can EDs integrate interventions into ED operations to increase feasibility and sustainability? Are existing staffing models sufficient to support the pragmatic implementation of interventions? | High priority  18 votes |
| How can interventions effectively leverage the EHR (e.g., the inclusion of ICD-10 does for social risks/needs in patient problem lists and EHR-facilitated interventions such as auto-referral lists)? | Low priority  6 votes |
| Gap 3: Engagement with medical and social systems | |
| How can interventions be tailored to increase patient linkage with resources and facilitate monitoring of outcomes? What forms of technology may be useful? | Mid priority  10 votes |
| Which interventions increase communication, coordination, and collaboration between EDs, their larger hospital or health systems, EMS, community partners, social services, and other systems? How can EDs provide warm handoffs to these systems? | Mid priority  11 votes |
